# Comparison of the 3D structures of mouse and human α-synuclein fibrils by solid-state NMR and STEM^☆^

**DOI:** 10.1016/j.jsb.2018.04.003

**Published:** 2019-04-01

**Authors:** Songhwan Hwang, Pascal Fricke, Maximilian Zinke, Karin Giller, Joseph S. Wall, Dietmar Riedel, Stefan Becker, Adam Lange

**Affiliations:** a Department of Molecular Biophysics, Leibniz-Forschungsinstitut für Molekulare Pharmakologie (FMP), 13125 Berlin, Germany; b Department of NMR-based Structural Biology, Max Planck Institute for Biophysical Chemistry, 37077 Göttingen, Germany; c Brookhaven National Laboratory, Upton, 11967 NY, USA; d Electron Microscopy Group, Max Planck Institute for Biophysical Chemistry, 37077 Göttingen, Germany; e Institut für Biologie, Humboldt-Universität zu Berlin, 10115 Berlin, Germany

**Keywords:** Solid-state NMR, Alpha-synuclein, Amyloid fibril, Protofilament, STEM

## Abstract

Intra-neuronal aggregation of α-synuclein into fibrils is the molecular basis for α-synucleinopathies, such as Parkinson’s disease. The atomic structure of human α-synuclein (hAS) fibrils was recently determined by Tuttle et al. using solid-state NMR (ssNMR). The previous study found that hAS fibrils are composed of a single protofilament. Here, we have investigated the structure of mouse α-synuclein (mAS) fibrils by STEM and isotope-dilution ssNMR experiments. We found that in contrast to hAS, mAS fibrils consist of two or even three protofilaments which are connected by rather weak interactions in between them. Although the number of protofilaments appears to be different between hAS and mAS, we found that they have a remarkably similar secondary structure and protofilament 3D structure as judged by secondary chemical shifts and intra-molecular distance restraints. We conclude that the two mutant sites between hAS and mAS (positions 53 and 87) in the fibril core region are crucial for determining the quaternary structure of α-synuclein fibrils.

## 1 Introduction

Aggregated α-synuclein is the main component of Lewy bodies, the pathological hallmark of Parkinson’s disease (PD) and other neurodegenerative disorders (Spillantini et al., 1998, Spillantini et al., 1997). Thus, determining the atomic structure of these aggregates represents a major goal in the field.

There are different polymorphic variants of α-synuclein fibrils that have been studied previously (Bousset et al., 2013, Dearborn et al., 2016, Heise et al., 2005), but a structure of full-length human α-synuclein (hAS) fibrils has only recently been presented (Tuttle et al., 2016). It was determined by solid-state NMR (ssNMR) spectroscopy – which is a valuable technique to gain insight into the atomic structures of amyloids and other supramolecular assemblies (Colvin et al., 2016, Lopez del Amo et al., 2012, Lu et al., 2013, Quinn and Polenova, 2017, Shi et al., 2015, Theint et al., 2017, Wälti et al., 2016, Xiang et al., 2017, Xiao et al., 2015) – in combination with scanning transmission electron microscopy (STEM).

An unexpected finding of this study was that the investigated hAS fibrils are composed of a single protofilament, as most amyloid fibrils are known to consist of multiple filaments. Here, we have investigated the quaternary structure (i.e. the number of protofilaments and interactions between protofilaments) of mouse α-synuclein (mAS) fibrils using a combination of ssNMR and STEM.

hAS and mAS possess 95% sequence similarity (Rochet et al., 2000) – their primary sequences differ at seven positions, with two mutant sites found in the fibril core region (residues ∼40 to ∼95): A53T and S87N (from hAS to mAS). Notably, the familial hAS mutant A53T has been linked to early-onset PD. This mutant aggregates more rapidly than wild-type hAS. Also mAS and hAS differ in their aggregation properties significantly: The human form aggregates slower than the mouse form and with a longer lag phase. Additionally, it is associated with PD in humans and in transgenic mice. Baum and coworkers have reported, that the A53T substitution dominates the aggregation growth rates, while the combination of the A53T and S87N mutations affects the lag phase of the aggregation of mAS compared to hAS (Kang et al., 2011). The other five mutation sites lie outside of the fibril core region (in the C-terminal region) and do not affect aggregation rates and lag phase significantly.

In order to gain further insights into the largely different aggregation kinetics, we set out to structurally characterize mAS fibrils and compare their structure with the structure of hAS fibrils.

## 2 Results and discussion

### 2.1 The same secondary structure with different numbers of protofilaments

**Fig. 1:**
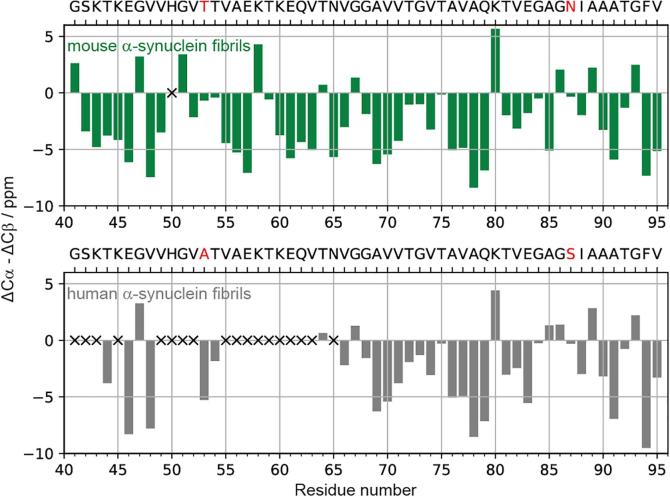
Secondary structure propensities of rigid cores of mouse (Lv et al., 2012) and human (Tuttle et al., 2016) α-synuclein fibrils. Secondary structure prediction by secondary chemical shifts (ΔCα-ΔCβ = (δCα_observed_ − δCα_random coil_) − (δCβ_observed_ − δCβ_random coil_)): positive, negative, and near-zero values are indicative of α-helix, β-strand and random coil regions, respectively. Missing chemical shift values are marked by a cross. The ΔCβ value of glycine is assumed to be 0. The two different mutant sites (residues at positions 53 and 87) are colored in red.

Notably, the mAS fibrils used in the present study exhibit the same secondary structure as the hAS fibrils previously used for the 3D structure determination (Lv et al., 2012, Tuttle et al., 2016), as judged by ^13^Cα and ^13^Cβ secondary chemical shifts (Luca et al., 2001) in Fig. 1. The observed pattern of positive and negative values of the secondary chemical shifts is remarkably similar, with the only notable difference found for residue A85. Therefore, it can be safely assumed that the mAS protofilament structure of our preparation is highly similar to the hAS protofilament structure of the preparation from Tuttle et al. (2016).

**Fig. 2:**
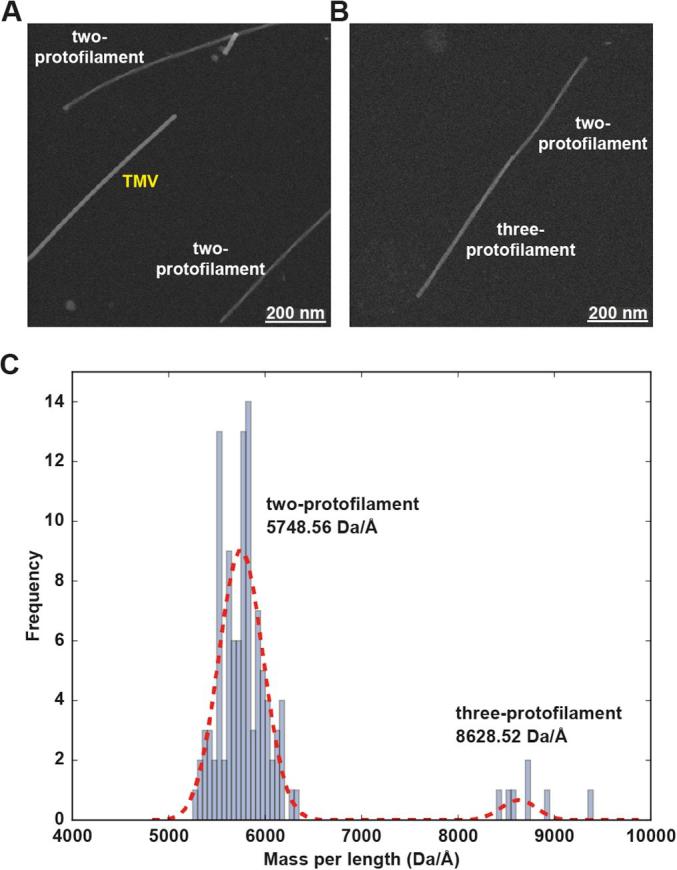
STEM analysis of unstained and freeze-dried mouse α-synuclein (mAS) fibrils. (*A*) Dark-field STEM image of tobacco mosaic virus (TMV) rods and mAS fibrils. TMV was used as an internal calibration standard (see also SI Appendix, Fig. S1). (*B*) Two or three protofilaments are axially adjoined in an mAS fibril. (*C*) Histogram of the mass-per-length (MPL) distribution for mAS fibrils. The MPL distribution was fitted using a bimodal Gaussian function as shown by the red dashed line. Values shown are means. Note that the MPL ratio of two- to three-protofilament fibrils is 2:3, exactly as expected.

On the primary structure level, only two residues are different between mAS and hAS in the core region of the fibril, comprising residues V40-V95: A53T and S87N. Despite the conservation of structure on the protofilament level, as seen by the secondary chemical shifts, surprisingly, the STEM data as presented in Fig. 2 reveal that mAS fibrils are composed of protofilament dimers (the predominant species) or even trimers (there was one incidence of a short single-protofilament – see SI Appendix, Fig. S1).

### 2.2 Weak protofilament-protofilament interactions

**Fig. 3:**
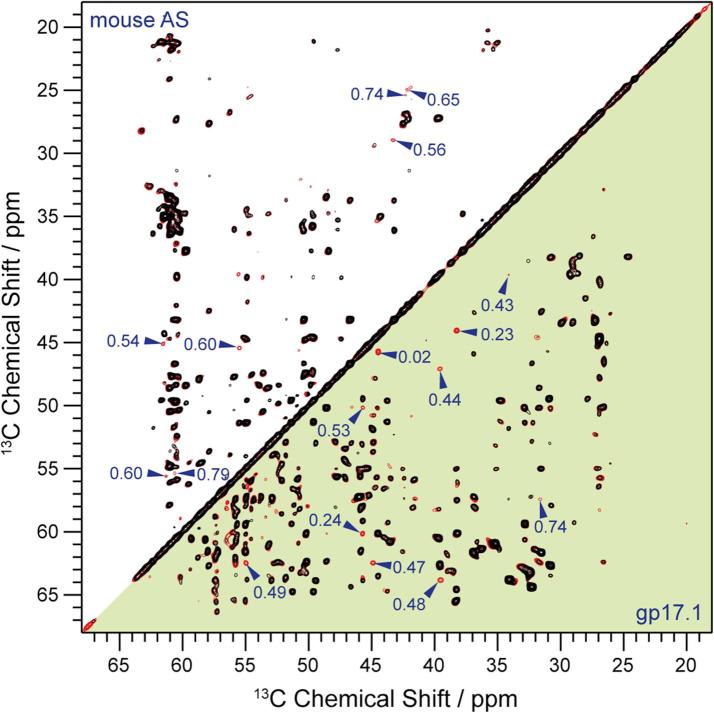
Comparison of long mixing time (500 ms proton driven spin diffusion, PDSD) ^13^C-^13^C 2D correlation spectra of mouse α-synuclein (mAS) fibrils (above diagonal) and assembled gp17.1 phage tail tube protein (below diagonal). Shown are the overlays of undiluted, uniformly ^15^N and [2-^13^C]-glycerol-labeled protein samples (red) and the corresponding diluted (1:3 labeled vs. unlabeled) samples (black). Red peaks not overlaid by black peaks represent inter-subunit contacts. For both samples, such peaks are annotated with the intensity ratio (diluted/undiluted). It can be seen that, for the mAS spectra, there are less peaks with sufficiently reduced intensity (ideally to 0.25) in the diluted spectrum. This lack of well-defined inter-molecular subunit restraints suggests that the interface between the subunits in the mAS fibrils is not identifiable with solid-state NMR and contrasts with other examples (in this case gp17.1 tail tubes).

Knowing that there are several protofilaments in the fibrils, we then set out to elucidate the inter-protofilament interface using ssNMR. For this purpose, we compared spectra of a non-diluted sample (all mAS molecules labeled) and a diluted sample (fibrils contain a random mixture of labeled and unlabeled mAS molecules in a ratio of 1:3). Intermolecular long-range correlations that could be used to elucidate the protofilament-protofilament interface should be identifiable by the fact that their corresponding cross-peak intensities would be reduced upon dilution by a factor of 4. Such peaks can, for example, be readily identified for the bacteriophage tail tube assembly that we used as a positive control (see Fig. 3, below diagonal) (Zinke et al., 2017). In contrast (Fig. 3, above diagonal), no such peaks are observable for mAS, even though the diluted spectrum was recorded with high signal to noise (total measurement time ∼3 weeks). The conclusion is that the interface between two mAS protofilaments must be small and/or hydrophilic in nature.

At this point it is interesting to compare our findings for mAS fibrils with the structures obtained for Aβ1–40 and tau fibrils. In both cases the number of protofilaments in the fibril has been determined to be two, in accordance with the number we found for the predominant species of mAS.

**Fig. 4:**
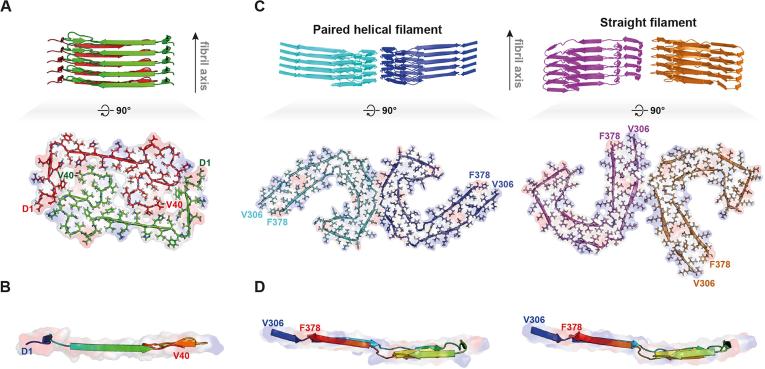
Structural comparison of Aβ1–40 E22Δ fibril (PDB entry 2MVX) (Schutz et al., 2015) and tau fibrils (PDB entries 5O3L for paired helical filaments (PHFs) and 5O3T for straight filaments (SFs)) (Fitzpatrick et al., 2017). Overviews of Aβ1–40 E22Δ fibril (*A*, upper) and polymorphic tau fibrils (*C*, upper) are shown in cartoon representation. The two protofilaments in the Aβ1–40 E22Δ fibril are associated laterally with no offset along the fibril axis, while the two protofilaments in PHFs and SFs are associated laterally with an offset of half a β-strand along the fibril axes. Top view (*A*, lower) and side view (*B*, only one monomer shown for clarity) of a layer in the Aβ1–40 E22Δ fibril, and top views (*C*, lower) and side views (*D*, only one monomer shown for clarity) of layers in the polymorphic tau fibrils are shown in cartoon and electrostatic potential surface representations. The degrees of monomeric extension along the fibril axes in Aβ1–40 E22Δ (*B*) and tau (*D*) fibrils are markedly different, i.e., the layer of Aβ1–40 E22Δ (*B*) is nearly flat, while the layers of tau fibrils (*D*) are extended along the fibril axes. The quaternary structure of tau fibrils would not be easily identifiable with solid-state NMR as the interface is very small (five amino acid residues for PHFs, 8–10 residues for SFs) and may involve the same residues in both protofilaments.

In the case of the Osaka mutant of Aβ1–40 (E22Δ), Meier and coworkers determined a high-resolution structure of the fibril using ssNMR and STEM (Schutz et al., 2015). Isotope-dilution experiments similar to the ones conducted by us clearly reveal the intricate and extensive hydrophobic interface between the two protofilaments (Fig. 4*A* and *B*). On the basis of our experiments, we can reject the presence of such an intricate interface in the case of mAS fibrils.

**Fig. 5:**
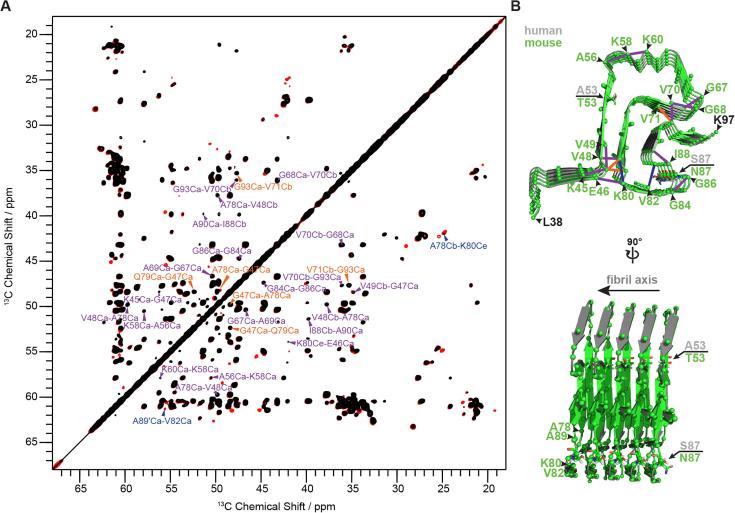
Intra- and intermolecular contacts in mouse α-synuclein (mAS) fibrils. (*A*) The same spectra are shown as in Fig. 3 (above diagonal) together with assignments: For intramolecular correlations, unambiguous medium-range (1 < |*i*-*j*| < 5) and long-range (|*i*-*j*| ≥ 5) restraints are annotated in orange. Further ambiguous restraints are assigned based on the atomic structure of human α-synuclein (hAS) fibrils (Tuttle et al., 2016) and are annotated in violet. Unambiguously assigned intermolecular restraints are annotated in blue. (*B*) Superposition of the human α-synuclein protofilament structure (PDB entry 2N0A) and the homology model of the mouse α-synuclein protofilament (see SI Appendix, Table S2). The assigned intra- and intermolecular correlations are indicated by solid lines following the same color code as in (*A*).

Recently, using cryo-EM, the structures of tau paired-helical filaments (PHFs) (Fig. 4*C* and *D*; left panel) and straight-filaments (SFs) Fig. 4*C* and *D*; right panel) have been determined at high-resolution (Fitzpatrick et al., 2017). It was shown that the protofilament structure is identical for PHFs and SFs, but that the protofilament-protofilament interfaces are different. In both cases the interfaces are relatively small, and rather hydrophilic. Such a type of interaction would indeed be compatible with the lack of clear intermolecular interactions observed by us for mAS. We note that the lack of these peaks could stem from the presence of a very polymorphic interface where the peak intensity of the long-range peaks would be scaled with the low population of the different states – however as the fibrils appear very ordered, as judged from the excellent resolution of the ssNMR spectra (see e.g. Fig. 5*A*), this does not seem very likely.

### 2.3 Similar protofilament fold between hAS and mAS

Although intra- and intermolecular correlations are not clearly distinguishable from the isotope-dilution experiment (Fig. 3, above diagonal), probable intra- and intermolecular correlations could be inferred from the differences in peak intensities between the spectra of the non-diluted sample and the diluted sample (see assignments in Fig. 5*A*). I.e. cross-peaks with similar peak intensities in both spectra are likely to be from intramolecular correlations (shown in orange and violet in Fig. 5*A* for unambiguous and ambiguous correlations, respectively) and cross-peaks with much weaker peak intensities in the diluted spectrum are more likely to be from intermolecular correlations (indicated in blue). The observed chemical-shift-unambiguous medium-range (1 < |*i*-*j*| < 5) and long-range (|*i*-*j*| ≥ 5) correlations are in good agreement with the reported structure of the hAS protofilament (Fig. 5*B*). Additionally observed ambiguous medium-range and long-range correlations are also in accordance with the hAS protofilament structure. Regarding probable intermolecular correlations, only two unambiguous correlations could be identified (A78Cb-K80Ce and A89′Ca-V82Ca, where the prime symbol indicates a signal from a second polymorph). They may result from a staggered arrangement of monomers along the fibril axis, similar to the arrangement found for tau fibrils (Fig. 4*D*), so that ^13^C-^13^C transfer between different neighboring molecules in the same protofilament may occur. A78 and A89 are tightly packed within the core region of mAS fibrils, so that intermolecular correlations between different protofilaments involving these residues would seem rather unlikely (Fig. 5*B*).

## 3 Conclusions

We conclude that for α-synuclein, similar to tau, protofilaments with the same 3D structure can assemble into various fibrils with a different number of protofilaments, e.g. one (hAS: previous study by Tuttle et al. (2016)); mAS: one incidence, see SI Appendix, Fig. S1), two (this study), or even three (this study, minor species). We note that some of the data presented by Tuttle et al. suggest the presence of dimeric hAS fibrils: In Fig. 4 of Tuttle et al. we have measured the width of TMV which is known to be close to 18 nm. In the graph, it is only 9 nm, indicating that the scale bar is too small by a factor of 2. Therefore, the width of the α-synuclein fibrils also needs to be multiplied by a factor of 2, yielding a value of ∼10–12 nm, which is in accordance with the value we observed for a *double*-protofilament fibril.

Since the intermolecular interfaces of mAS fibrils are not as intricate and extensively hydrophobic as for Aβ1–40 (E22Δ), for which an excellent high-resolution ssNMR structure could be determined, cryo-EM will be the method of choice to determine the ultimate quaternary structure of mAS fibrils and reveal the basis of the various protofilament-protofilament interfaces. Due to the fact that mAS and hAS differ in the fibril core region only at two positions, and as their protofilament fold is highly similar, it is likely that these residues play a key role for determining the quaternary structure.

## 4 Materials and methods

### 4.1 Sample preparation

Samples of mouse α-synuclein (mAS) fibrils were prepared as described previously (Lv et al., 2012). Samples of assembled gp17.1 phage tail tube protein were prepared as described previously, except for the deuteration (Zinke et al., 2017). The samples used in the present study were protonated. The labeled and undiluted samples were recombinantly expressed in *E. coli* in a medium containing [2-^13^C]-glycerol and [^15^N]-ammonium chloride as sole carbon and nitrogen sources, respectively. The diluted (1:3 labeled vs. unlabeled) samples were prepared by mixing labeled and unlabeled protein in a ratio of 1:3 before polymerization. The samples (see SI Appendix, Fig. S2) were packed with a few crystals of DSS (4,4-dimethyl-4-silapentane-1-sulfonic acid) into 4 mm ZrO_2_ magic-angle spinning (MAS) rotors.

### 4.2 Mass-per-length measurements

Dark-field STEM images of 10- and 30-fold diluted, unstained, freeze-dried mAS fibrils were recorded at Brookhaven National Laboratory, USA. Mass-per-length (MPL) measurements of mAS fibrils and tobacco mosaic virus (TMV) rods were conducted using “Amyloid-2” (an integral box with a width of 28 nm and a length of 74 nm) and “TMV” (an integral box with a width of 20 nm and a length of 36 nm) models in the PCMass 3.2 software (Wall and Simon, 2001) (available at ftp://ftp.stem.bnl.gov). A selection of images that contain TMV MPL near an average of 13.1 kDa/Å with a standard deviation of <2.32% resulted in 9 images out of 64. All MPL values of mAS fibril segments in the selected images (111 segments in total) were adjusted by calibrating the TMV MPL values to the known value of 13.1 kDa/Å. The method of non-linear least squares was used to fit a bimodal Gaussian function to a histogram of MPL distribution for mAS fibrils. Data fitting and visualization were performed using Matplotlib (Hunter, 2007), SciPy and NumPy (van der Walt et al., 2011).

### 4.3 Solid-state NMR spectroscopy

Two-dimensional (2D) ^13^C-^13^C correlation spectra (500 ms proton driven spin diffusion, PDSD) of mAS fibrils and assembled gp17.1 phage tail tubes were acquired on an NMR spectrometer with 800 MHz ^1^H Larmor frequency (Bruker Biospin, Germany) and a 4 mm [^1^H, ^13^C, ^15^N]-triple channel MAS probe. All measurements were conducted at an MAS frequency of 11 kHz and a sample temperature of 5–7 °C. The sample temperature was monitored by the temperature dependent chemical shift position of the water peak with the DSS peak calibrated to 0 ppm (Bockmann et al., 2009), and the ^13^C/^15^N chemical shifts were calibrated with the DSS as an internal reference (Markley et al., 1998). The 2D ^13^C-^13^C PDSD spectra were obtained via an initial ^1^H 90° pulse, ^1^H-^13^C ramped cross-polarization (CP) (Hediger et al., 1995, Metz et al., 1994) followed by ^13^C-^13^C spin diffusion. SPINAL-64 ^1^H decoupling (Fung et al., 2000) was applied during ^13^C evolution and detection periods. The detailed experimental parameters are summarized in SI Appendix, Table S1. The acquired spectra were processed with Bruker TopSpin 3.2 and analyzed using CcpNmr 2.4.2 (Stevens et al., 2011) and SPARKY version 3.1 (T. D. Goddard and D. G. Kneller, University of California).

### 4.4 Protofilament modeling

Homology models of a 5-meric mAS protofilament were built using MODELLER (Sali and Blundell, 1993) based on the determined human α-synuclein (hAS) structure (PDB entry 2N0A) (Tuttle et al., 2016). mAS and hAS have the same length of 140 amino-acid residues and share 95% identity with seven mutations (from hAS to mAS: A53T, S87N, L100M, N103G, A107Y, D121G and N122S). In order to obtain a template structure of hAS for the homology modeling, five monomers out of the 10-meric hAS protofilament (Tuttle et al., 2016) were extracted using PyMOL 1.3 (http://www.pymol.org/). In the template structure of hAS, the flexible N-(residues 1–37) and C-termini (residues 98–140) were discarded to build a homology model of the core region only. Ten homology models of a 5-meric mAS protofilament were calculated based on the template structure of hAS with the variable target function method (VTFM) (Braun and Go, 1985), followed by evaluation of the ten models using MolProbity (Chen et al., 2010). The best model out of the ten models was selected based on the MolProbity score, MODELLER objective function score, and DOPE score (see Fig. 5*B* and SI Appendix, Table S2).

### 4.5 Molecular visualization

Atomic structures of Aβ1–40 (E22Δ) fibrils, tau fibrils, hAS fibrils and mAS homology model were visualized utilizing PyMOL 1.3 (http://www.pymol.org/).
