# Supplementary material for: Comparison of the 3D structures of mouse and human α-synuclein fibrils by solid-state NMR and STEM
Source: J Struct Biol. 2019 Apr 1;206(1):43–8. doi: 10.1016/j.jsb.2018.04.003 (PMC6470123; doi:10.1016/j.jsb.2018.04.003)
Supplement: Supplementary data [file mmc1.docx]

**Supporting Information for**

Comparison of the 3D Structures of Mouse and Human α-Synuclein Fibrils by Solid-State NMR and STEM

Songhwan Hwang^a^, Pascal Fricke^a^, Maximilian Zinke^a^, Karin Giller^b^, Joseph S. Wall^c^, Dietmar Riedel^d^, Stefan Becker^b^, and Adam Lange^a,e,1^

^a^Department of Molecular Biophysics, Leibniz-Forschungsinstitut für Molekulare Pharmakologie (FMP), 13125 Berlin, Germany; ^b^Department of NMR-based Structural Biology, Max Planck Institute for Biophysical Chemistry, 37077 Göttingen, Germany; ^c^Brookhaven National Laboratory, Upton, 11967 NY, USA; ^d^Electron Microscopy Group, Max Planck Institute for Biophysical Chemistry, 37077 Göttingen, Germany; ^e^Institut für Biologie, Humboldt-Universität zu Berlin, 10115 Berlin, Germany.

^1^To whom correspondence should be addressed. Telephone: +49 30 947 93 190. E-mail: [alange@fmp-berlin.de](mailto:alange@fmp-berlin.de).


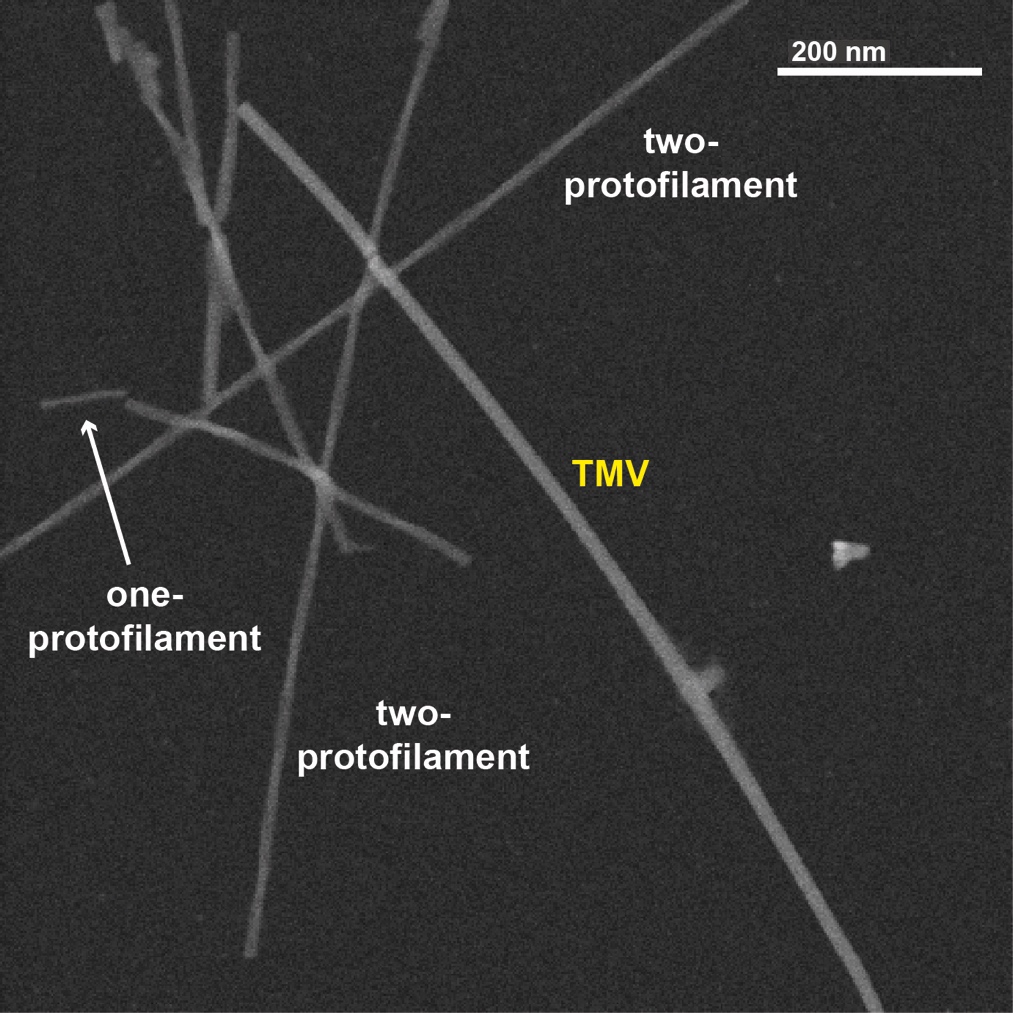


Fig. S1. STEM analysis of unstained and freeze-dried mouse α-synuclein (mAS) fibrils. Dark-field STEM image of tobacco mosaic virus (TMV) rods and mAS fibrils. TMV was used as an internal calibration standard. A one-protofilament fibril was observed only once in all the STEM images.


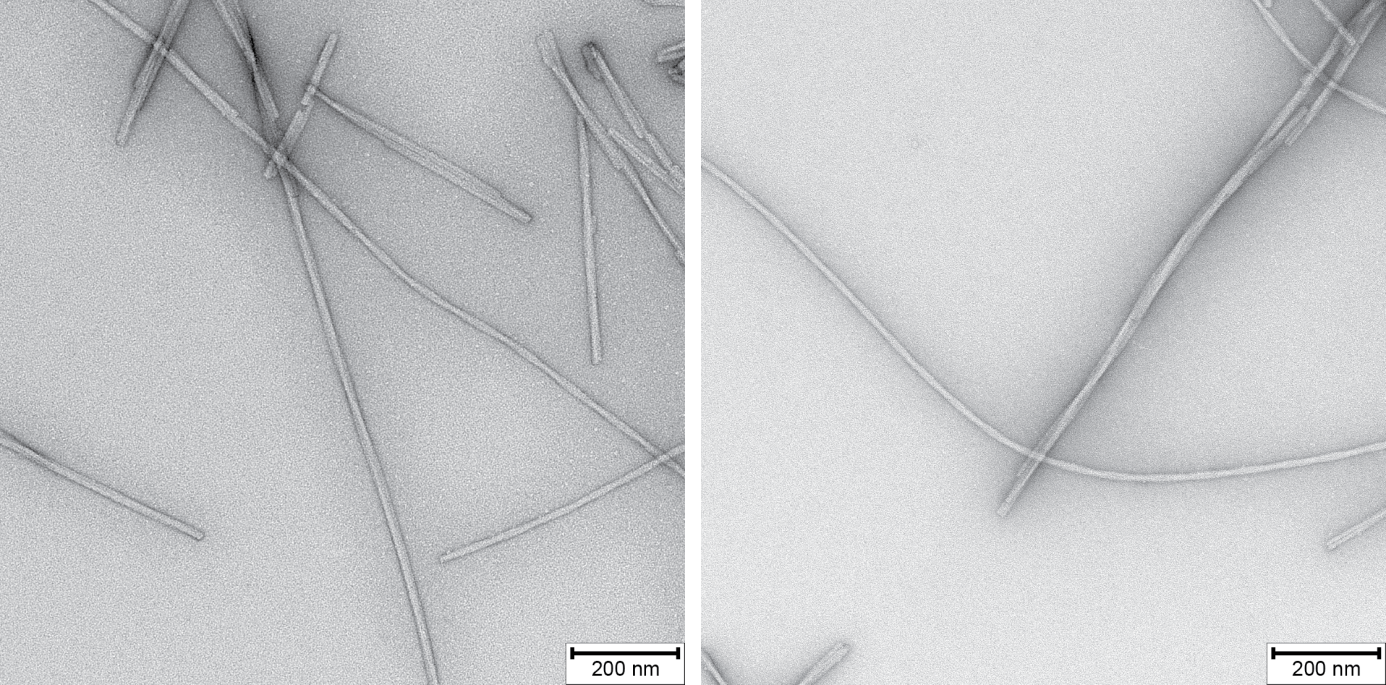


Fig. S2. Transmission electron microscopy micrographs of mouse α-synuclein (mAS) fibrils. Undiluted, uniformly ^15^N and [2-^13^C]-glycerol-labeled protein sample (left panel) and diluted (1:3 labeled vs. unlabeled) sample (right panel).

Table S1. Set of pulse program parameters used for the acquisition of the 2D ^13^C-^13^C correlation spectra of mouse α-synuclein (mAS) fibrils and assembled gp17.1 phage tail tubes at 800 MHz ^1^H Larmor frequency and 11 kHz magic-angle spinning. The radio frequency (r.f.) power values were only determined for the high-power pulses via nutation experiments, the other r.f. powers are calculated assuming a linear amplifier and are therefore only estimates. The same parameters were used for undiluted (^15^N and [2-^13^C]-glycerol-labeled) and diluted (1:3 labeled vs. unlabeled) samples.

| **Parameter** | **Value for mAS** | **Value for gp17.1** |
| --- | --- | --- |
| Recycle delay | |  |
| Recycle delay | 2 s | 2 s |
| 90° initial ^1^H excitation pulse | |  |
| R.f. power | 83.33 kHz | 83.33 kHz |
| Duration | 3 µs | 3 µs |
| ^1^H-^13^C CP step | |  |
| ^1^H r.f. power | 74.26 kHz | 75.04 kHz |
| ^13^C r.f. power | 50 kHz | 50 kHz |
| Ramp shape | 80-100 % on ^1^H | 80-100 % on ^1^H |
| Duration | 1.4 ms | 1.4 ms |
| ^13^C evolution time | |  |
| SPINAL-64 r.f. power | 83.33 kHz | 83.33 kHz |
| SPINAL-64 pulse duration | 5.8 µs | 6.5 µs |
| Maximum acquisition time | 14 ms | 12 ms |
| 90° ^13^C flip pulses | |  |
| R.f. power | 50 kHz | 50 kHz |
| Duration | 5 µs | 5 µs |
| ^13^C-^13^C spin diffusion | |  |
| Mixing time | 500 ms | 500 ms |
| Acquisition | |  |
| SPINAL-64 r.f. power | 83.33 kHz | 83.33 kHz |
| SPINAL-64 pulse duration | 5.8 µs | 6.5 µs |
| Acquisition time | 14 ms | 15 ms |
| Total experimental time |  |  |
| Undiluted | 2 days and 15 hours | 2 days and 22 hours |
| Diluted | 23 days and 5 hours | 15 days and 14 hours |

Table S2. Scores for the ten homology models of 5-meric mAS protofilament.

| **Model** | **MolProbity Score** | **MODELLER objective function score** | **DOPE score** |
| --- | --- | --- | --- |
| 1 | 2.61 | 1659.59 | -24938.39 |
| 2 | 2.79 | 1704.84 | -25014.03 |
| *3 | 2.54 | 1600.52 | -25160.36 |
| 4 | 2.71 | 1791.84 | -24581.90 |
| 5 | 3.00 | 1855.24 | -24969.74 |
| 6 | 3.10 | 1802.57 | -24723.89 |
| 7 | 2.66 | 1828.26 | -24953.59 |
| 8 | 2.85 | 1717.62 | -24706.27 |
| 9 | 2.59 | 1660.90 | -25157.50 |
| 10 | 2.61 | 1650.17 | -24964.25 |

*The selected best model
